# Critical incidents in a tertiary care clinic for internal medicine

**DOI:** 10.1186/1756-0500-6-276

**Published:** 2013-07-16

**Authors:** Paula Scharein, Marten Trendelenburg

**Affiliations:** 1 Clinic for Internal Medicine, University Hospital Basel, Petersgraben 4, CH, 4031 Basel, Switzerland

**Keywords:** Critical incidents, CIRS, Communication, Medication

## Abstract

**Background:**

Reducing medical errors has become an international concern. Population-based studies consistently demonstrate inacceptable high rates of medical injury and preventable deaths. Thus, electronic critical incident reporting systems are now increasingly used in hospitals, predominantly in anesthesia. However, studies systematically analyzing critical incidents are scarce. Our aim was to describe content and causes of critical incidents in our Clinic for Internal Medicine.

**Results:**

We retrospectively analyzed all critical incidents reported during a 54-months period. Between implementation and analysis, 456 incidents were reported anonymously in the commercially available platform-independent, web-based critical incident reporting system. All incidents were analyzed according to the reporting profession, time point during hospitalization process, content and potential causes.

Most incidents occurred on medical wards (80%). The most frequent type of incidents was medication errors (62%). These incidents primarily occurred when prescribing and/or administering drugs (30% and 29% of medication errors respectively). So-called, human errors’, i.e. occurring without apparent external factor, were the most frequently indicated cause of critical incidents (56%) followed by insufficient communication (26%). These problems primarily occurred between different groups of health care professionals and between different departments. The described types and reasons of critical incidents remained stable during the observation period.

**Conclusions:**

The findings of our analysis of the character and type of critical incidents occurring in a tertiary care clinic for internal medicine reported in an anonymous, voluntary, electronic reporting system suggest that strategies to improve communication and medication delivery are most promising to avoid critical incidents.

## Background

Population-based studies from a number of nations around the world consistently have demonstrated unacceptably high rates of medical injuries and preventable deaths [1]. Thus, reducing medical errors has become an international concern. One option to achieve this goal is the systematic reporting and analysis of critical incidents being defined as unintentional events that endangered the patient, but did not harm [2]. Systems to report critical incidents may help to unmask structures and processes leading to potentially harmful medical errors and as a consequence might prevent medical injuries. A critical incident reporting systems (CIRS) was firstly described as an outgrowth of studies in the *Aviation Psychology Program* of the *United States Army Air Forces* in World War II [3]. This program was established in summer 1941 to develop procedures for the selection and classification of aircrews [4]. Later it was used for the NASA’s *Aviation Safety Reporting system (ASRS)* in 1976. In 1971 *Blum* was the first who transferred this method of analysis to anesthesia [5] and consecutively for the first time to medicine. In the following, more expanded studies were performed by *Cooper* et al. in 1978 and later again in 1984 who analyzed the main incidents in anesthesia [6,7]. *Leape et al.* demonstrated that a large fraction of critical incidents occurring in *New York Hospitals* could be preventable [8] leading to the conclusion that a critical incident reporting system (CIRS) could measurably improve patient safety [9].

Nowadays, electronic critical incident reporting systems are more and more used in hospitals, predominantly in anesthesia [10,11].

However, data analyzing the content and type of incidents are still scarce, in particular in clinics with non-invasive treatment strategies such as general internal medicine. Thus, the aim of the presented study was a systematic analysis of all critical incidents having been reported in our clinic for internal medicine in Basel in order to identify major fields for improvements. The entries were made during a period of four and a half years between April 2007 and October 2011. To the best of our knowledge this is the first analysis of unselected critical incidents occurring in a tertiary care clinic for general internal medicine.

## Methods

The clinic for internal medicine at the *University Hospital in Basel* takes care of the majority of internal medicine inpatients of our hospital that are pooled in our clinic. All specialties of internal medicine are involved. The electronic CIRSmedical® recorded all incidents having occurred and been reported at any time of the hospitalization process between admission in the emergency room and discharge to home or to another institution such as a surgical clinic, a rehabilitation clinic or a home for senior citizens. The electronic system allows separating critical incidents according to the place of occurrence since every department (surgery, internal medicine, dermatology, neurology, anesthesia, gynecology, ORL, laboratories, radiology and ophthalmology) maintains its own system (so-called CIRS subunits). Thus, critical incidents being analyzed in this study were limited to those being classified as “internal medicine”. In addition, due to the separation into CIRS subunits our analysis mostly excluded critical incidents having occurred on an intensive care unit.

CIRSmedical® is a commercially available, web-based, platform-independent critical incident reporting system provided by *ProtecData AG, Oberwil, Switzerland*. It was initiated by the *Perioperative Patient-Safety Grou*p at the *University of Basel* in Switzerland collaborating with NASA psychologists. This led to the first CIRS used in anesthesia at the University Hospital of Basel. This initial system was developed further to CIRSmedical® that now can be used in clinical medicine in general. CIRSmedical® was implemented in our clinic in April 2007. It fulfills all criteria characteristic of a successful reporting system [9]: Non-punitive, confidential, independent, system-oriented and timely analyzed by an expert group, i.e. the so-called CIRS board. The CIRS board in our clinic consists of experienced medical doctors and nurses, who are capable of disseminating and implementing recommendations into the clinical processes. Besides the analysis of incidents and the implementation of recommendations, the CIRS board also selects reports that by mistake were reported by the wrong department and makes sure that these reports are shifted to the correct unit.

Critical incidents were defined as any undesirable but preventable event having occurred in the care of patients that might have led or indeed led to a medical error. Thus, critical incidents analyzed in this study predominantly consisted of so-called *near misses* but also included reports of *adverse events*.

The analyzed incidents were reported between implementation of the system in April 2007 and October 2011. In total 456 incidents were recorded and analyzed. Reported incidents were continuously evaluated by the CIRS board followed by a retrospective categorization in a predefined classification sheet by a trained clinical research fellow. The categorization was confirmed by the head of the CIRS board.

Primarily four main objectives were analyzed: The reporting person, the content, the cause and the point in time during hospitalization. In addition, comments written by anonymous health care professionals and feedbacks by the CIRS board were evaluated. Entries predominately were made by nursing staff and physicians but in principal could be made by any person being involved in the medical process of our hospital.

The contents were categorized to bleeding complications, medication related errors, incidents occurring in the context of an intervention, problems due to failure of apparatus/computers, mixing-up of patients, incidents occurring in the context of a clinical trial or in the context of a chemo-/radiotherapy. Multiple categories per incident were allowed. Reports on incidents in medication related incidents were further classified in wrong dosage, wrong prescription, mixing up of medication (wrong drug to correct patient), mixing up of patients (correct drug but administered to the wrong patient), drug not received or not categorisable.

The causes were classified into problems due to insufficient communication, errors in documentation and/or transmission of information (on paper), so-called human errors (defined as incidents in which either no external factor could be identified or in which the reporting person itself declared an absence of external factors), instrument and/or computer problems, excessive labor and/or tiredness, shortage of staff, and incidents due to stress/multitasking/diversion. Incidents with failures in communication were further classified to problems occurring among nursing staff, among physicians, between nursing staff and physicians/other health care professionals, between health care professionals and patients, or between different departments, e.g. in the context of a patient transfer from one clinic to another one.

All critical incidents in our system were made anonymous. The incidents remained in the original order but precise dates could not be identified any more. In an attempt to analyze changes in character of critical incidents over time, the data set was divided into groups consisting of 100 incidents each. Considering a total number of n=456 and an overall period of four and a half years, each group of 100 incidents probably comes close to a period of about one year. This procedure was supported by the judgment of the CIRS board that had continuously followed all cases such that it confirmed that the rate of incidents per months during the whole period was not undergoing major changes.

Parameters were compared using Chi-square tests or in case of small numbers by Fisher’s exact test. All testing was two-tailed, and p values less than 0.05 were considered to be statistically significant. All statistical analyses were performed using Prism for Windows, version 4 (GraphPad).

## Results

Eighty percent of the critical incidents happened during hospitalization. Sixty-one percent were reported by nursing staff and 35.5% by a physician. During the four and a half-year period increasing relative numbers of incidents were reported by nursing staff (49% of the first 100 incidents were reported by nursing staff versus 77% of the incidents at the end of the study period; p<0.0001). The number of comments varied during the different periods between 32 and 98 per 100 incidents and between 0 (first hundred incidents) and 20% of incidents were commented by the CIRS board.

**Figure 1:**
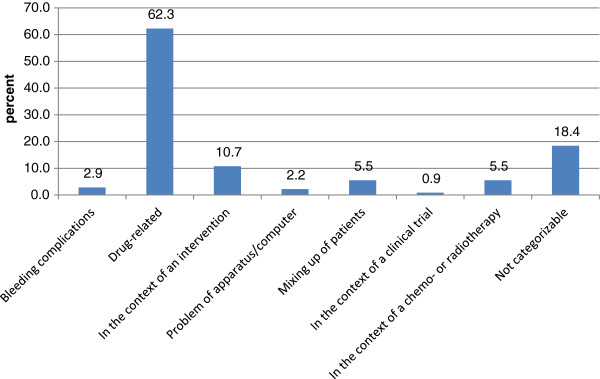
The content of critical incidents reported (n=456).

The most frequent content of the reported incidents (shown in Figure 1) were drug related errors (62.3%). Less frequent incidents could not be categorized (18.4%), occurred in the context of an intervention (10.7%), dealt with mixing up of patients (5.5%) or occurred in the context of chemo- and/or radiotherapy (5.5%). Less than 5% of reported incidents were bleeding complications, problems with an apparatus/computer or occurred in the context of a clinical trial. Overall, the contents were not found to underlie major changes over time (not shown).

**Figure 2:**
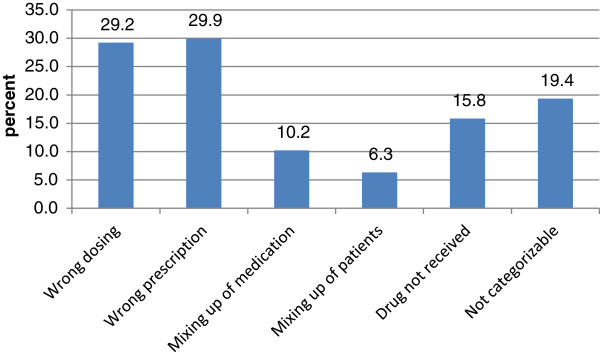
**Subgroup analysis of drug-related errors (n=315).** Wrong prescriptions and wrong dosage were reported most frequently.

Since drug-related errors predominated, these incidents were further classified (Figure 2). Thirty percent of drug-related incidents were wrong prescriptions and 29% referred to a wrong dosage. In 19.4% of reports the incidents could not be categorized, in 15.8% of incidents the patient did not receive the medication, in 10.2% of incidents the medication was mixed up and in 6.3% of incidents the patient receiving the drug was mixed up.

**Figure 3:**
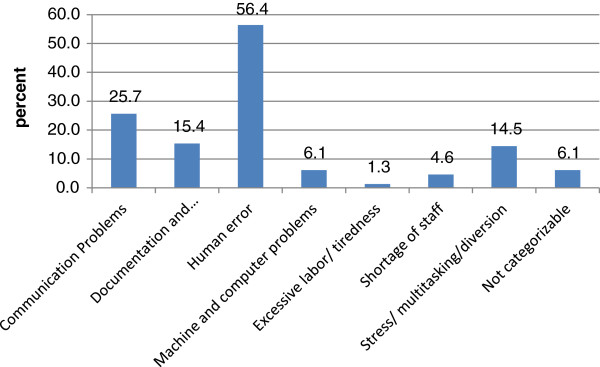
The causes of reported critical incidents (n=456).

The most frequent cause of critical incidents (Figure 3) were so-called ‘human errors’, i.e. occurring without apparent external factor (56.4%). Less frequent were communication problems (25.7%), documentation and transmission errors (15.4%), stress/multitasking/diversion as cause of the incident (14.5%), machine and/or computer problems (6.1%) or not categorisable incidents. Less than 5% of incidents mentioned shortage of staff or excessive labor/ tiredness. Overall, the causes of critical incidents were not found to underlie major changes over time (not shown).

**Figure 4:**
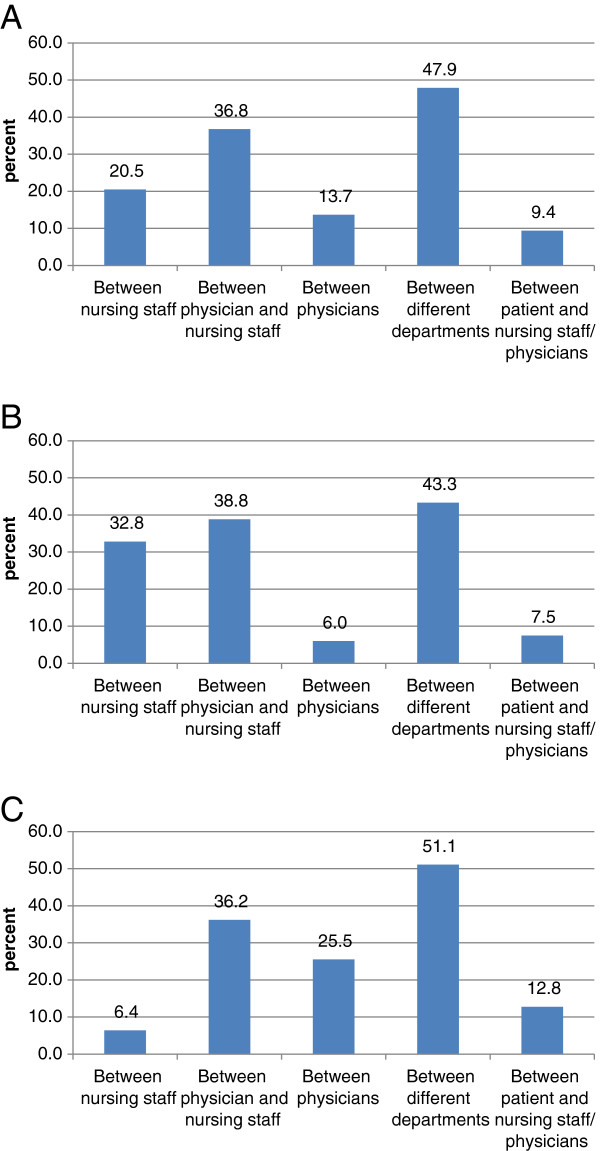
**Subgroup analysis of communication errors (n=150, A), and comparison of communication problems reported by nursing staff (n=86) (B) versus those reported by physicians (n=62) (C).** Nursing staff as well as physicians both primarily reported on incidents having occurred between different departments as a major cause of incidents followed by incidents due to insufficient communication between physician and nursing staff.

A more specific look at the communication problems (Figure 4A) showed that they occurred in 47.9% of incidents between different departments, in 36.8% between physician and nursing staff, in 20.5% between nursing staff, in 13.7% between physicians and in 9.4% between patient and nursing staff/ physicians.

**Figure 5:**
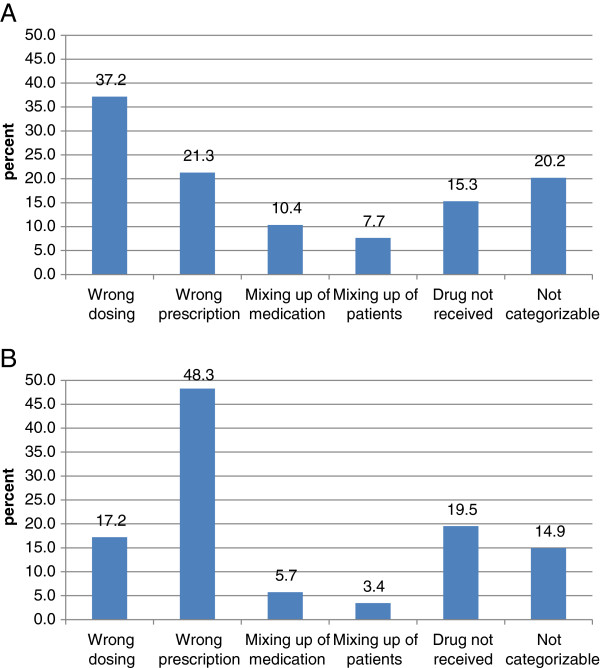
**Comparison of drug related errors reported by nursing staff (n=205) (A) versus those reported by physicians (n=95) (B).** Nursing staff reported more errors in wrong dosage of drugs (37.2%) than physicians (37.2% versus 17.2%, p=0.0025) and less with a wrong prescription (21.3% versus 48.3%, p<0.0001).

Finally, we compared incidents reported by physicians with those reported by nursing staff. Causes of incidents reported by these two groups of health care professionals were similar (data not shown; p=0.48). Interestingly, in terms of communication problems, nursing staff as well as physicians both primarily reported incidents having occurred between different departments as a major cause of incidents followed by incidents due to insufficient communication between physician and nursing staff (Figure 4B + C). In terms of content, both groups of health care professionals reported most frequently on drug-related errors (66.1% by nursing staff versus 53.7% by physicians; not shown). However, among these drug-related errors nursing staff reported more wrong dosages of drugs than physicians (37.2% versus 17.2%, p=0.0025) and less wrong prescriptions (21.3% versus 48.3%, p<0.0001) being in line with the processes in our clinic in which drugs are prescribed by medical doctors but prepared and administered by nursing staff (Figure 5A + B). Among all critical incidents including non-drug related errors, nursing staff more frequently reported mixing-up of patients (7.6% versus 1.2% by physicians, p=0.035) and errors having occurred in the context of a chemo- or radiotherapy (8.3% versus 1.2%, p=0.035) whereas physicians reported more frequently bleeding complications (7.4% versus 0.4% by nursing staff, p=0.0068) and more frequently incidents that could not be categorized (27.8% versus 13.7%, p=0.0147).

## Discussion

To our knowledge this is the first description of the character of critical incidents occurring in a tertiary care clinic for general internal medicine that were reported in an anonymous, voluntary, non-punitive, confidential and independent electronic reporting system.

The most frequent type of incidents reported were medication errors, especially errors in prescription and dosing. The two main causes of critical incidents described were so-called human errors, i.e. errors without apparent or well defined external factor, and communication failures, particularly between physicians and nursing staff and between different departments.

With these results there are different starting points for preventing critical incidents. Whereas addressing ‘human errors’ is likely to be difficult since this type of incident was very heterogeneous ([11], data not shown) any effort leading to a reduction in medication problems (e.g. double checking of the administered drugs [12] or an electronic prescription, order and delivery of drugs) might lead to a significant reduction in medical critical incidents occurring on internal medicine wards. This observation is well in line with studies by Bates et al. on the frequency of adverse drug events in hospitalized adult patients [13,14].

In addition we identified shortcomings in communication between different groups of health care professionals and between different departments/clinics as a major cause of critical incidents. Whereas communication between physicians and patients is recognized as an important source of misunderstanding and consequently of potential treatment errors [14-16] our data suggest that insufficient communication between health care professionals and between medical departments is not only a strain for the health care professionals [17,18] but also needs to be recognized as important source of critical incidents in medicine [19-22]. In addition, insufficient communication seems to be a concern for both, physicians and nursing staff.

According to the number of comments by anonymous readers and the feedbacks given by the CIRS board we assume that the electronic incident reporting system has been accepted and is in regular use by many of our health care professionals. However, as a limitation of our study the reported incidents that were analyzed in our study cannot be considered as being complete and/or representative for all critical incidents occurring in our clinic. In addition, in one study a routine incident reporting system was found to be poor at identifying patient safety incidents, particularly those resulting in harm [23]. However, in the study by *Sari et al.*, special form sheets needed to be filled and sent to a local reporting system where the incidents were classified and entered into the database. In contrast, the faster and easier reporting of new incidents in our electronic CIRS, an important strength of our study, might have increased the routine use and thus facilitated the recognition of processes provoking medical errors. Independently, an electronic CIRS has the major advantage of surveying routine parameters of reporting errors at places or situations where they occur and where concerned health care professionals voluntarily decided to report on the event. Thus, in spite of important limitations we think that an electronic CIRS as analyzed in our study is an excellent tool to uncover relevant problems in the medical care of patients.

Further limitations of our study were its retrospective character and the fact that the analysed data derived from a single center. The retrospective character mostly limited our possibility of more detailed analyses of the reported incidents since the electronic template used for reports had not specifically been designed for this purpose. In addition, due to the single center character it remains uncertain to which extent our observations can be transferred to other hospitals. The strongly interdisciplinary care in our clinic in which general internists regularly interact not only with specialists of the sub-disciplines of internal medicine but also with medical doctors of non-internal medicine clinics might have significantly increased the fraction of communication problems and therefore differ from highly specialized clinics with rather limited interdisciplinary character. However, as observed in our analysis, we think that the increasing specialization that can be observed in medicine indeed is leading to increasing conflicts due to insufficient communication in particular between groups of health care professionals as well as between different medical clinics.

Independently, a major problem and challenge for future studies is the limited evidence that implementation of a CIRS in the daily hospital routine indeed helps preventing critical incidents and/or improves patient care. Few studies have attempted to address this question. *Valentin et al.* could show that the number of errors in medication (wrong drug, wrong dose, wrong route) was lower in hospitals with a CIRS compared to hospitals without [24]. The identification of outcome measures is a major obstacle, taking into account that the implementation of CIRS might affect unpredicted parameters and that the definition of a control period should only differ by the implementation of CIRS without additional measures to reduce medical errors. In an attempt to address the question whether the introduction of CIRSmedical® in our clinic indeed led to a reduction in medical accidents we compared the number of liability cases since implementation of CIRSmedical® (2007 to 2011) with the corresponding period before, i.e. between 2002 and 2006. Interestingly, liability cases since implementation of CIRSmedical® decreased to less than half of the number of the control period. However, as outlined above, the documented cases are influenced by a number of factors and therefore cannot be directly linked to the implementation of CIRSmedical® in our clinic.

## Conclusion

In conclusion, critical incidents occurring in our tertiary care clinic for internal medicine that were reported in an anonymous electronic reporting system were found to predominantly consist of medication errors. The most frequently indicated causes of critical incidents were so-called ‘human errors’, i.e. errors without apparent and/or well defined external factor, and insufficient communication, in particular between physicians and nursing staff as well as between different departments. As a consequence, we think that strategies to improve communication and the process of medication seem to be the most promising to avoid critical incidents in internal medicine.

## Competing interests

The authors declare that they have no competing interests.

## Authors’ contributions

PS acquired, analysed and interpreted the data, was involved in drafting and revising the manuscript and gave final approval of the version to be published. MT designed the study and was involved in analysis and interpretation of data. He was involved in drafting the manuscript and revising it critically for important intellectual content. He also gave final approval of the version to be published. PS and MT, both take public responsibility for appropriate portions of the content. Both authors read and approved the final manuscript.
